# Functional diversity of resilin in Arthropoda

**DOI:** 10.3762/bjnano.7.115

**Published:** 2016-09-01

**Authors:** Jan Michels, Esther Appel, Stanislav N Gorb

**Affiliations:** 1 Department of Functional Morphology and Biomechanics, Institute of Zoology, Christian-Albrechts-Universität zu Kiel, Am Botanischen Garten 1–9, D-24118 Kiel, Germany

**Keywords:** biological materials, biomechanics, composites, elastomeric proteins, functional morphology

## Abstract

Resilin is an elastomeric protein typically occurring in exoskeletons of arthropods. It is composed of randomly orientated coiled polypeptide chains that are covalently cross-linked together at regular intervals by the two unusual amino acids dityrosine and trityrosine forming a stable network with a high degree of flexibility and mobility. As a result of its molecular prerequisites, resilin features exceptional rubber-like properties including a relatively low stiffness, a rather pronounced long-range deformability and a nearly perfect elastic recovery. Within the exoskeleton structures, resilin commonly forms composites together with other proteins and/or chitin fibres. In the last decades, numerous exoskeleton structures with large proportions of resilin and various resilin functions have been described. Today, resilin is known to be responsible for the generation of deformability and flexibility in membrane and joint systems, the storage of elastic energy in jumping and catapulting systems, the enhancement of adaptability to uneven surfaces in attachment and prey catching systems, the reduction of fatigue and damage in reproductive, folding and feeding systems and the sealing of wounds in a traumatic reproductive system. In addition, resilin is present in many compound eye lenses and is suggested to be a very suitable material for optical elements because of its transparency and amorphousness. The evolution of this remarkable functional diversity can be assumed to have only been possible because resilin exhibits a unique combination of different outstanding properties.

## Review

### Resilin – the pliant protein

**Figure 1:**
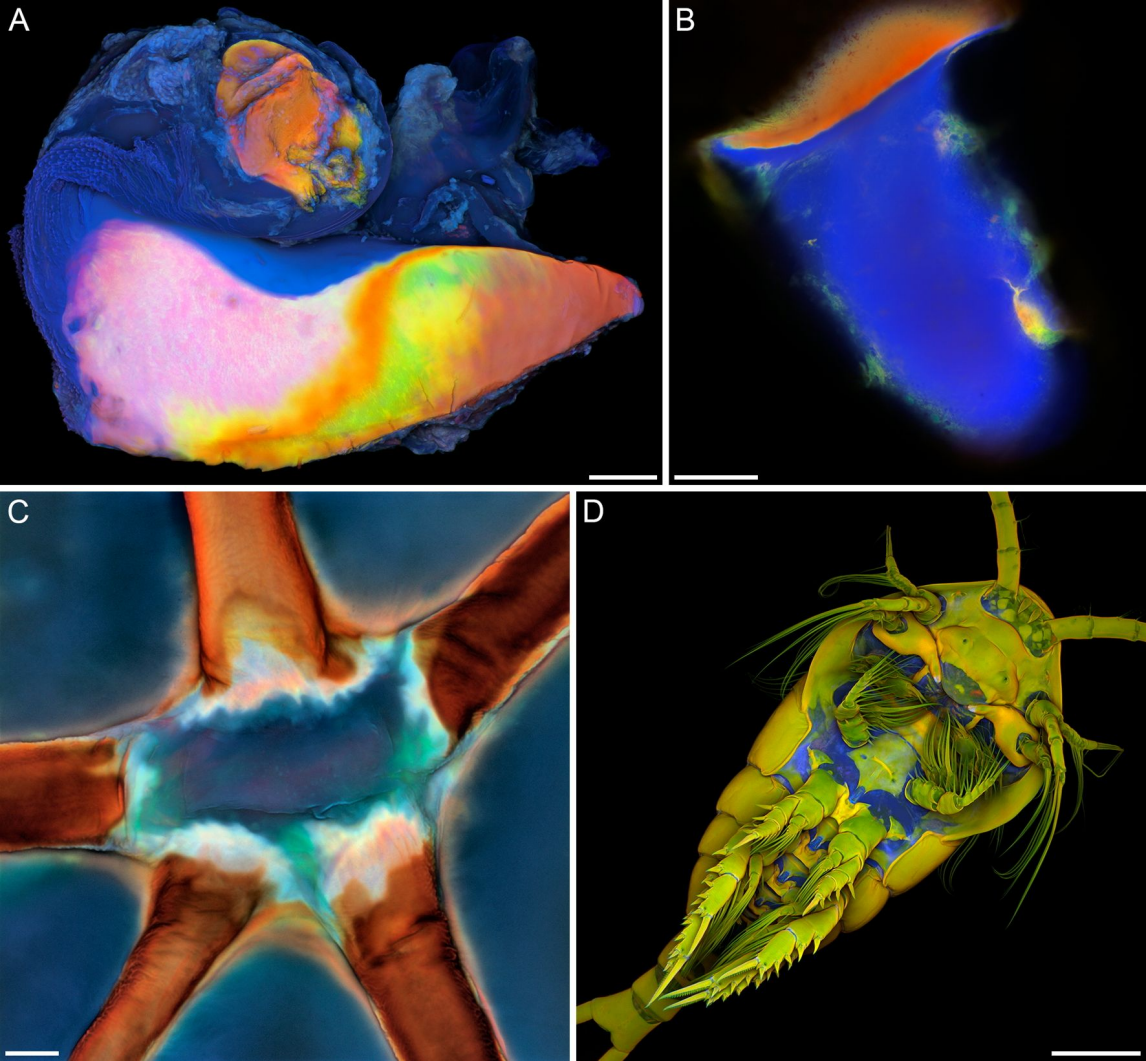
Occurrence of resilin in insects and crustaceans. Confocal laser scanning micrographs showing a wing hinge (A) and a prealar arm (B) of the migratory locust (*Locusta migratoria*), a wing vein joint of the common darter (*Sympetrum striolatum*) (C) and the ventral side of a female copepod of the species *Temora longicornis* (D). (A–C) Overlays of four different autofluorescences exhibited by the exoskeleton. Blue colours indicate large proportions of resilin, while green structures consist mainly of non- or weakly-sclerotised chitinous material, and red structures are composed of relatively strongly sclerotised chitinous structures. (D) Blue = autofluorescence of resilin, red = Congo red fluorescence of stained chitinous exoskeleton parts, green = mixture of autofluorescence and Congo red fluorescence of stained chitinous exoskeleton parts. (A, C, D) Maximum intensity projections. (B) Optical section. Scale bars = 100 µm (A, B), 20 µm (C), 200 µm (D). (A–D) Adapted with permission from [10], copyright 2011 John Wiley and Sons.

Elastomeric proteins occur in a large range of organisms and biological structures, and the spectrum of their biological functions is very broad [1]. They feature a great diversity including well-known examples such as elastin, titin and fibrillin present in vertebrate muscles and connective tissues, byssus and abductin of bivalve molluscs and gluten of wheat [1]. Besides spider silk proteins, resilin is certainly the best-known among the elastomeric proteins existing in arthropods. The first description of resilin, which has often been called rubber-like protein, was based on analyses of three different insect exoskeleton elements: the wing hinge and the prealar arm of the desert locust (*Schistocerca gregaria*) (also described for the migratory locust (*Locusta migratoria*), Figure 1A,B) and the so-called elastic tendon of the pleuro-subalar muscles in dragonflies of the genus *Aeshna* [2]. Additional insights into the characteristics of resilin that had been gained shortly after this description [3–4] resulted in a comprehensive compilation of the then existing knowledge of resilin properties [5]. Resilin consists of a network of randomly orientated coiled polypeptide chains that have a high degree of flexibility and mobility and are linked together at regular intervals by stable covalent cross-links. Only the fully cross-linked protein is called resilin, whereas the not yet cross-linked or not fully cross-linked protein is called pro-resilin [6]. Within hydrolysates of resilin, glycine constitutes the largest proportion (30–40%) of the total residues [7–8]. Such hydrolysates also feature the two unusual amino acids dityrosine and trityrosine, which were identified to form the cross-links between the polypeptide chains [9].

The mechanical properties of resilin strongly depend on the degree of hydration because resilin is plasticised by water [5]. When resilin is completely hydrated, it behaves close to a perfect rubber [3–4,11]. Due to its molecular prerequisites, resilin then exhibits a near-perfect resilience of up to 92–97% and a fatigue limit of over 300 million cycles [12]. With respect to resilience, resilin is unmatched by any other elastomeric protein and the best synthetic rubbers such as unfilled polybutadiene [13–14]. Fully hydrated resilin has a rather low stiffness. In the elastic tendons of dragonflies and locust ligaments mentioned above, it was found to have a Young’s modulus of 0.6–0.7 MPa and 0.9 MPa, respectively [11]. In addition, fully hydrated resilin can be stretched to more than three times its original length and compressed to one third of its original length, and when the tensile and compressive forces are released, resilin goes back to its initial state without having any residual deformations [3–4,13].

Until today, resilin has been found to exist mainly in insect exoskeleton structures where this protein has a number of different functions, which include (1) the storage of elastic energy in jumping systems [15–20], (2) the reduction of fatigue in folding wings of beetles and dermapterans [21–22], (3) the enhancement of the adaptability of attachment pads to uneven surfaces [23] and (4) the generation of flexibility of wing vein joints in dragonflies and damselflies [24–26]. Resilin has also been reported to be present in the exoskeletons of other arthropod taxa such as crustaceans [10,27–30] (Figure 1D), scorpions [31] and centipedes [32]. In addition, resilin-like proteins that contain dityrosine and trityrosine are known to exist in several non-arthropod taxa including monogeneans [33–34], nematodes [35], mussels [36] and sea urchins [37] indicating that resilin likely originated much earlier in the evolution of invertebrates than previously assumed.

The properties of resilin-containing exoskeletons can strongly differ between structures and organisms. The reason is that in biological structures resilin seems to be rarely present in pure or nearly pure form but is known to commonly exist together with other proteins and/or chitin fibres in resilin-containing composites, which exhibit a mixture of the properties of the single components. In such composites, the resilin properties can even be ‘overlain’ by the properties of the other components making an identification of the presence of resilin in the respective structures with the criteria of Andersen and Weis-Fogh [5] very difficult. In addition, certain exoskeleton structures feature only some of the typical characteristics of resilin-containing material but lack the others. It is then often not possible to determine whether these structures contain resilin or other proteins resembling resilin. In such cases, it is conceivable that the respective exoskeleton material consists either of a protein with properties that are similar to those of resilin or of a mixture of resilin and other proteins. For exoskeleton structures with such properties, the term ‘transitional cuticle’ was established [5].

In order to allow a classification of exoskeleton structures as resilin-containing exoskeleton according to the definition of Andersen and Weis-Fogh [5], these structures must conform to the wing hinge, the prealar arm and the elastic tendon mentioned above with respect to their properties, which can be tested with a number of methods [5]. Resilin is colourless, transparent and amorphous. Accordingly, structures with very large resilin proportions can be easily distinguished from structures with relatively large proportions of chitin that are typically pigmented and only slightly transparent or sometimes, when the sclerotisation is very pronounced, not transparent at all. When immersed in aqueous media and in many anhydrous hydrophilic liquids, resilin exhibits an isotropic swelling, which is reversible and depends on the pH. (It is least pronounced at pH values of about 4.) In its hydrated state, resilin is swollen and features its typical rubbery nature, long-range deformability and complete elastic recovery. Furthermore, if hydrated resilin is tensioned, it will become birefringent, and the birefringence will be positive in the direction of the extension. When resilin is completely dried, it loses its rubber-like characteristics and becomes relatively hard and brittle. Proteolytic enzymes such as pepsin or trypsin can be applied to test for the presence and distribution of resilin, because resilin is known to be digested by such enzymes. Resilin has been shown to be stained by single conventional dyes. Chemical reactions with the Masson and Mallory dyes were mentioned to stain resilin red. Staining of resilin with aqueous solutions of methylene blue and toluidine blue is a common method and can provide good information about the presence and distribution of resilin. When resilin is stained with one of these two dyes, it does not show metachromasia. Among the amino acids that form resilin, dityrosine and trityrosine exhibit a relatively pronounced autofluorescence. This autofluorescence is present in natural resilin-containing structures and in isolated resilin (both before and after boiling in water) and in resilin hydrolysates. In neutral and alkaline solutions, its excitation and emission maxima are at about 320 nm and 415 nm, respectively [38–39]. The excitation spectrum of the resilin autofluorescence differs with changing pH conditions. In acid solutions, the excitation maximum is shifted considerably to about 285 nm, and the upper edge of the excitation peak is at about 330–340 nm [38–39]. The pH-induced changes of the excitation properties are reversible and take place rapidly [40].

The described resilin identification and visualization methods are not absolutely specific. Therefore, it is strongly advisable to apply not only one single method but a combination of several different ones to increase the reliability of the identification and detection of resilin. In recent years, an antibody to a recombinant *Drosophila melanogaster* pro-resilin (rec1-resilin) was developed and has been shown to be cross-reactive and to label resilin in different insects [12–13,41–42]. Until today, this immunohistochemical method has been tested for only a small number of insects and only within the studies mentioned above. If it proves efficient in tests with a larger number of arthropod species, it will represent the first reliable method that specifically identifies resilin.

The development and improvement of methods applying techniques such as micromechanical testing, atomic force microscopy and confocal laser scanning microscopy (CLSM) have facilitated detailed studies of the distribution, composition and mechanical properties of resilin-containing exoskeleton structures in diverse organisms and at different levels of their organisation. One of these methods utilises a combination of different autofluorescences. In addition to resilin, other arthropod exoskeleton materials also exhibit autofluorescences, which can be efficiently visualized with fluorescence microscopy. This allows the production of overlays consisting of different micrographs that show different autofluorescences. Such overlays nicely exhibit differences in the autofluorescence composition, which are good indications for differences in the material composition and clearly reveal structures with relatively large resilin proportions within the analysed specimens [21,23,43]. However, when analysing arthropod exoskeleton structures for the presence of the autofluorescence of resilin, one has to bear in mind that some other compounds present in organisms exhibit autofluorescences whose properties are similar to those of the resilin autofluorescence, with excitation maxima in the UV range and emission maxima in the violet and blue ranges of the light spectrum [44–47].

If the autofluorescences are visualized by means of CLSM, the results will be very detailed and precise [10]. With a recently described method, four different autofluorescences are excited and detected separately, and certain colours are allocated to each of the four visualized fluorescence signals. On the resulting overlays, exoskeleton structures with large proportions of resilin are blue, while green structures consist mainly of non- or weakly-sclerotised chitinous material, and red structures are composed of relatively strongly sclerotised chitinous structures (for details see [10]; Figure 1A–C). Many of the confocal laser scanning micrographs shown in this review were created using this method.

**Figure 2:**
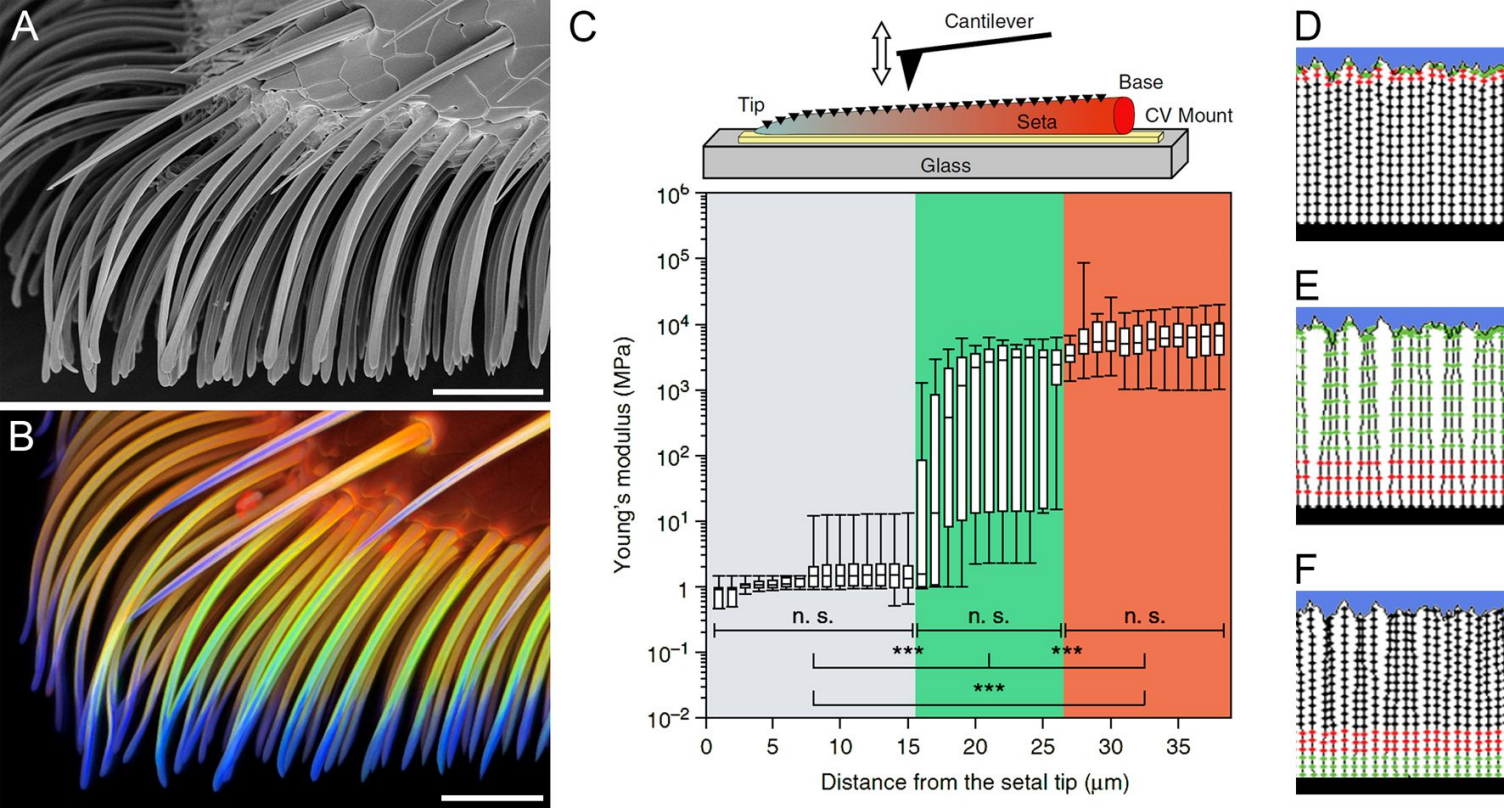
Distribution, mechanical properties and functional significance of resilin in adhesive tarsal setae. (A, B) Ventral part of the second adhesive pad of a female seven-spot ladybird (*Coccinella septempunctata*), lateral view. (A) Scanning electron micrograph. (B) Confocal laser scanning micrograph (maximum intensity projection) showing an overlay of four different autofluorescences exhibited by the exoskeleton. Blue colour indicates the presence of large proportions of resilin. (C) Box-and-Whisker plots showing the median Young’s modulus of fresh adhesive tarsal setae obtained by atomic force microscopy nanoindentations (see the inset above the graph) along each seta. The borders of the boxes define the 25th and 75th percentiles, the median is indicated by a horizontal line, and the error bars define the 10th and 90th percentiles (n. s. = not significant, *** = highly significant). The background colours indicate the different seta sections. (D–F) Numerical model showing typical configurations of a filamentary structure (setal array) attached to a stiff support (black rectangle) in adhesive contact with a random fractal surface (blue region). Three types of fibres were tested: (D) stiff fibres with short elastic ends, (E) long elastic fibres connected to the base by short stiff roots and (F) stiff fibres with soft elastic segments near the base. The different stiffnesses of the segments are conditionally shown by circles with different colours. Stiff, medium and soft segments are marked by black, red and green circles, respectively. Scale bars = 25 µm (A, B). (A–C) Adapted with permission from [48], copyright 2013 Nature Publishing Group. (D–F) Adapted with permission from [49].

Very often, gradients of the material composition with a considerably changing proportion of resilin are present in arthropod exoskeleton structures. Such resilin proportion gradients must also be reflected by gradients of the mechanical properties of the respective resilin-containing composites. The material composition of adhesive tarsal setae of beetles (Figure 2B) represents a good example for such gradients. Recently, the Young’s modulus of such setae was measured along the longitudinal axis of the setae (Figure 2C). The measurements revealed that the Young’s modulus of the material in the most distal section of each seta is relatively low (1.2 ± 0.3 MPa), whereas it is considerably higher at the setal base (6.8 ± 1.2 GPa). The differences in the Young’s modulus between different regions correlate with the resilin proportion observed in the seta material [48]. When the setae are dehydrated, the Young’s modulus of the setal tip material strongly increases from 1.2 to 7.2 GPa, and it exhibits no statistically significant differences along the complete setae [48], which is in accordance with the relationship between the material properties and the hydration status of resilin mentioned above. Besides the differences in the Young’s modulus, the mechanical behaviour of the respective materials shows the pronounced differences in the material composition between the tips and the bases of fresh adhesive tarsal setae. While the material of the tip features only elastic deformation, both elastic and, to some extent, plastic deformation are observed in the material of the base [48]. This means that the purely elastic response of the tip is due to the presence of resilin, whereas the partially plastic deformation at the base is mainly due to the presence of stiffer tanned exoskeleton. It is very likely that effects similar to those observed in beetle adhesive tarsal setae exist in other exoskeleton structures with comparable gradients of the resilin proportion.

### Occurrence and functions of resilin in different arthropod exoskeleton systems

Resilin is known from numerous arthropod exoskeletons where it is present in diverse structures and allows manifold functions, which in most cases are based on its very pronounced elasticity and its ability to completely recover after deformation. For example, resilin plays an important role in flight systems of insects, in particular in insects that use a wing beat with a low frequency (10–50 Hz) (see below). Resilin-containing exoskeleton structures have been described for various mechanical systems including leg joints [40,50], vein joints and membranous areas of insect wings [21–22,24], the food-pump of reduviid bugs [51], tymbal sound production organs of cicadas [52–53] and moths [54], abdominal cuticle of honey ant workers [55] and termite queens [56], the fulcral arms of the poison apparatus of ants [57] and the tendons of dragonfly flight muscles and basal wing joints of locusts (as already mentioned above) [5]. In the following, some selected representative structures and systems with large proportions of resilin are highlighted, and their functions are described.

#### Arthrodial membranes

Arthrodial membranes are cuticle areas that are typically thin, non-sclerotised and very flexible. Such membranes often are multifunctional units. The soft cuticles of caterpillars, for example, have a combination of both a protective and a locomotory role, which is reflected in their ultrastructural architecture [58]. The main functions of arthrodial membranes are to connect sclerotised exoskeleton elements and allow relative movement of these elements and to extend whenever an increase in volume of the body is necessary [59–60]. In addition, some membranes are armoured with miniature protuberances on their surfaces and have a defence function [61–62]. For insects, two different types of membranes have previously been reported. The first type is a highly extensible membrane found in the locust abdomen that can extend up to ten times its original length [63–65]. This cuticle is highly specialised in its protein composition [66]. The second type is a folding laminated membrane that is less stretchable and has been found, for example, in the abdomen of the tsetse fly *Glossina morsitans* [66] and in the bug genus *Rhodnius* [67–68].

**Figure 3:**
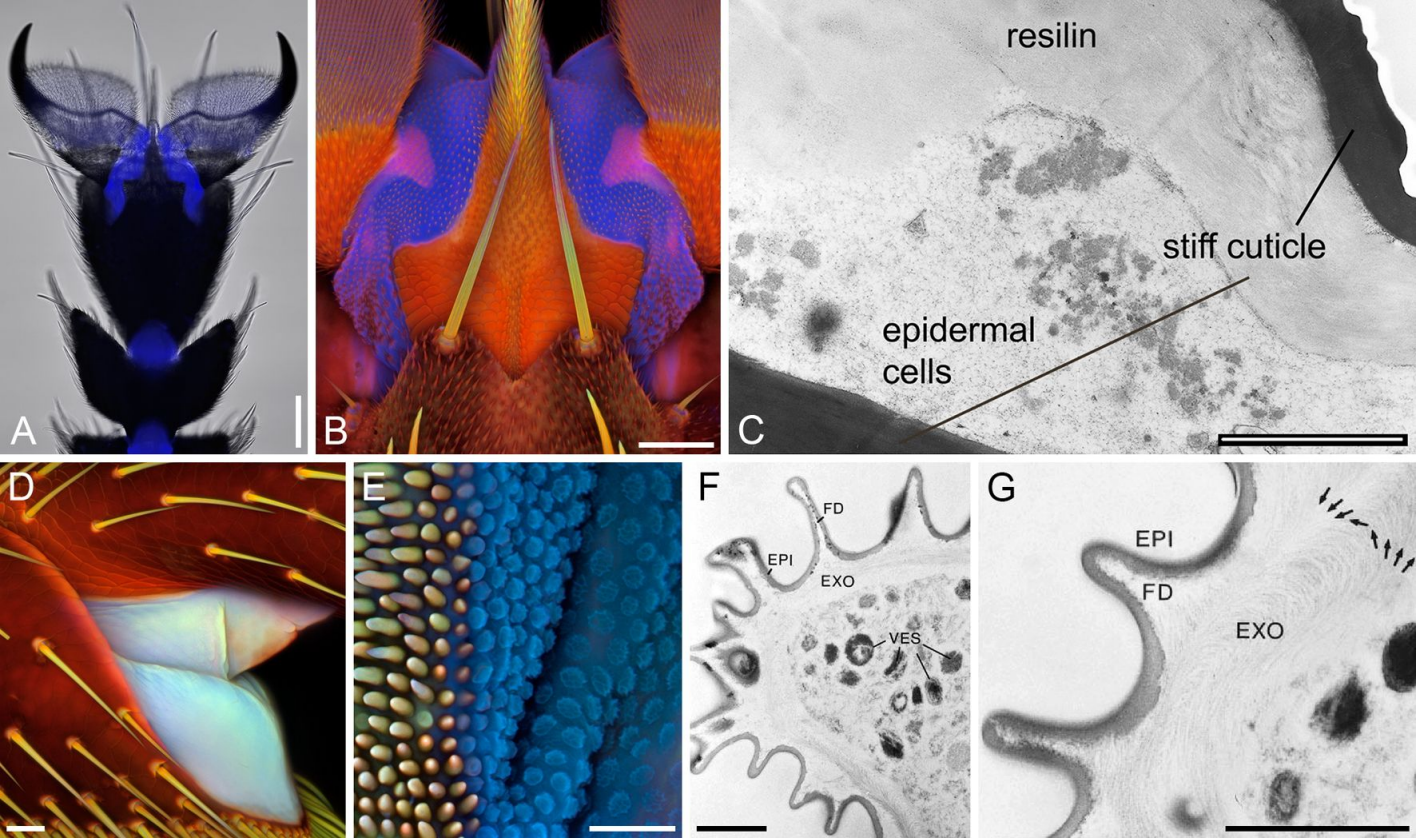
Resilin in arthrodial membranes of insects. (A, B) Pretarsus of the drone fly (*Eristalis tenax*), ventral view. (A) Overlay of a bright-field micrograph and a wide-field fluorescence micrograph showing the presence and distribution of resilin autofluorescence (blue). (B) Confocal laser scanning micrograph (maximum intensity projection) revealing large proportions of resilin (shown in blue) in the membranous structures between rigid sclerites of the pretarsus. (C) Pretarsus of the urban bluebottle blowfly (*Calliphora vicina*). Transmission electron micrograph showing an ultra-thin section through structures comparable to those shown in blue in B. (D) Lateral view of the joint between the tarsomeres 1 and 2 in the third leg of the seven-spot ladybird (*Coccinella septempunctata*). (E) Border between the neck membrane (right side) and a postcervical sclerite (left side) of the broad-bodied chaser (*Libellula depressa*). (F, G) Membranous cuticle in the neck area of the blue-tailed damselfly (*Ischnura elegans*). Transmission electron micrographs showing ultra-thin sections. EPI, epicuticle; EXO, exocuticle; FD, folds; VES, vesicles. Arrows: preferential orientation of chitin fibres. Scale bars = 100 µm (A), 50 µm (B), 2 µm (C), 20 µm (D, E), 1 µm (F, G). (A, B, D, E) Adapted with permission from [10], copyright 2011 John Wiley and Sons. (F, G) Adapted with permission from [69], copyright 2000 The Zoological Society of London.

Membranous cuticle often contains large proportions of resilin (Figure 3). Examples are membrane structures connecting claws and pulvilli to the terminal tarsomere [43,50]. In the pretarsus of the drone fly (*Eristalis tenax*) (Insecta, Diptera, Syrphidae), for example, membranous cuticle with large proportions of resilin forms a spring-like (or joint-like) element (Figure 3A–C) that makes the pulvilli movable and thereby enables them to efficiently adapt to the substrate. In general, joints in legs typically feature membranes, which often contain large proportions of resilin and allow the relative movement of the joint elements (Figure 3D).

The neck membrane of dragonflies is another example. This flexible cuticle connects the neck sclerites and enables an extensive mobility of the head [69]. A recent study clearly revealed that the neck membrane material of the broad-bodied chaser (*Libellula depressa*) contains relatively large proportions of resilin, while the neighbouring sclerites are mainly composed of sclerotised chitinous material [10] (Figure 3E). Transmission electron microscopy showed that dragonfly neck membrane cuticle is rather homogenous and electron-lucent [69]. Membranous areas of insect cuticle nearly always exhibit a relatively intensive autofluorescence similar to that of resilin. This suggests that arthrodial membranes generally contain relatively large proportions of resilin. However, even very soft and flexible membranes such as the dragonfly neck membrane do not consist of pure resilin but rather represent resilin–chitin composites in which some reinforcement by chitin-bearing microfibrils is clearly visible (Figure 3F,G).

#### Legged locomotion

Mechanisms of fast leg movements with an acceleration that can surpass the limitations of muscle contraction have been found in different insect groups including fleas [15,70], locusts [71], beetles [72–73] and true bugs [16–18,74]. The respective catapult-like devices have often evolved to enhance the acceleration in relatively short legs [75]. They usually contain specific types of joints that are typically supplemented with active power or latch muscles producing tractive force and trigger muscles that are responsible for releasing elastic energy from specific energy storage devices (see below).

**Figure 4:**
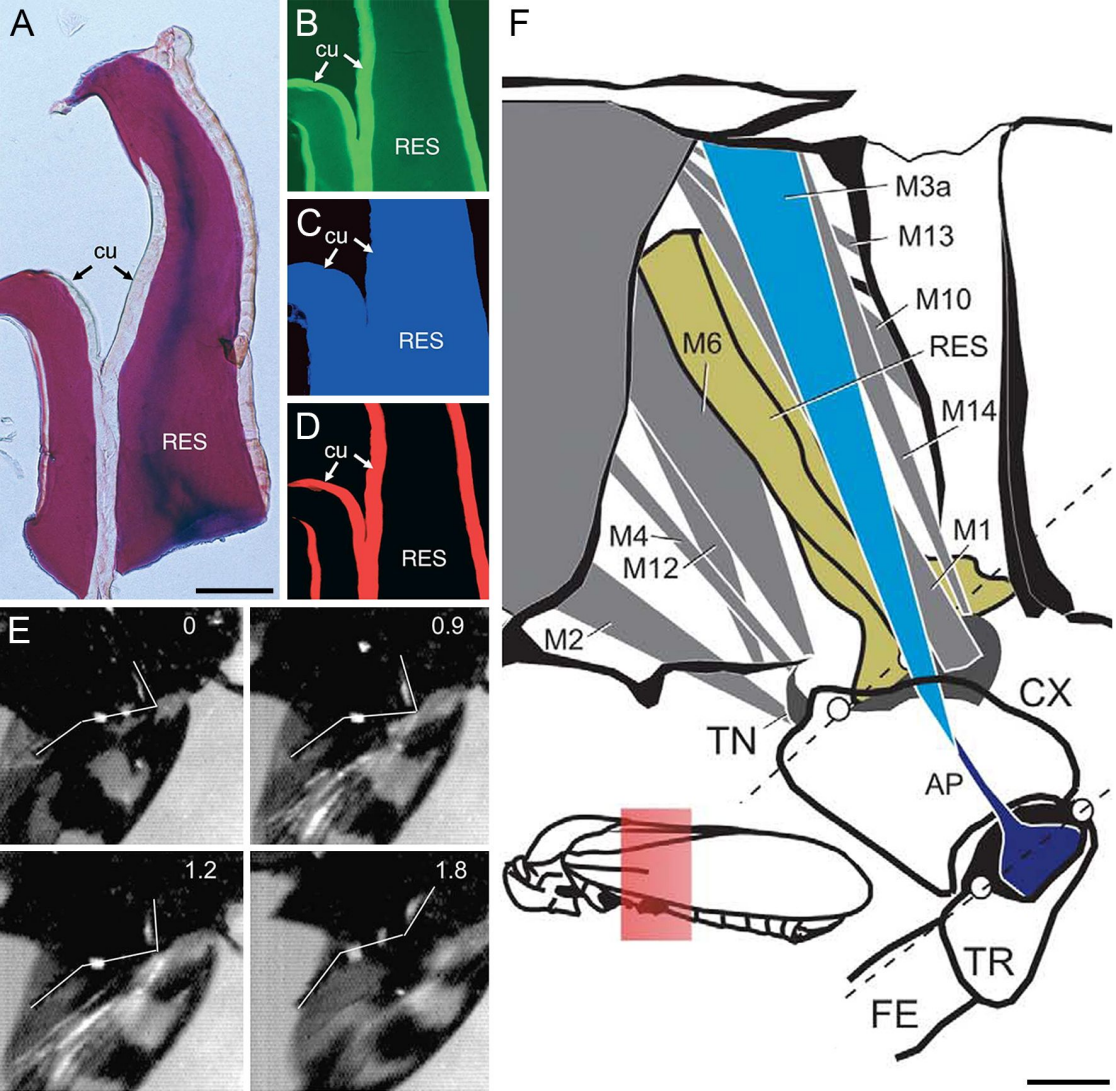
Resilin in the jumping system of the black-and-red froghopper (*Cercopis vulnerata*). (A–D) Cuticle supplemented with resilin in the pleural area of the metathorax. Horizontal sections through the pleural seam. (A) Paraffin-embedded cuticle stained after Cason. Bright-field micrograph. (B–D) Frozen sections of the structures shown in A. Wide-field fluorescence micrographs showing different autofluorescences exhibited by the exoskeleton. (B) Excitation: 512–546 nm; emission: 600–640 nm. (C) Excitation: 340–380 nm; emission: 420 nm. (D) Excitation: 710–775 nm; emission: 810–890 nm. cu, tanned cuticle; RES, structures containing resilin. (E) Sequence of single frames of a high-speed video recording of the cicada jump (ventral aspect). The numbers indicate the time scale in milliseconds. The approximate positions of femur, tibia and tarsus are indicated by white lines according to white marker dots on the leg. (F) Skeleton-muscle organisation of the cicada metathorax (medial aspect, right side). AP, apodeme of the trochanter extensor muscle M3; CX, coxa; FE, femur; M1, M2, M4, M6, M14, subcoxal muscles; M7, M9, M10, M11, M12, M13, M14, metathoracic muscles; M5, trigger muscle; M8a, M8b, trochanter flexor muscle; RES, resilin; TN, trochantine; TR, trochanter; white circles, condyli of the coxa and trochanter. The inset shows the position of the structures in the entire insect body. (A–F) Adapted with permission from [16], copyright 2004 Elsevier.

In Auchenorrhyncha and Sternorrhyncha, the jumps are performed by metathoracic muscles that are directly connected to the trochanter of the hind leg and responsible for the movements of both trochanter and coxa [16,74] (Figure 4F). In the jumping cicada called black-and-red froghopper (*Cercopis vulnerata*) (Cercopidae), the complete extension of the hind leg takes less than one millisecond [16] (Figure 4E). This suggests that, in addition to the muscle system, an elastic spring system powers the jump. The application of fluorescence microscopy and histological staining revealed structures with large proportions of resilin in the pleural area of the metathorax (Figure 4A–D). These structures stretch dorso-ventrally across the entire pleural area (Figure 4F) and are much larger than comparable structures present in fleas (see below). Their dorsal and ventral parts are located close to the origin of the lateral portion of the power muscle and closely connected to the lateral part of the coxa, respectively. The resilin-containing structures very likely participate in the extension of both coxa and trochanter by the release of energy that is stored by deflection or twisting of their bar-like shape.

Fleas possess two pads with large proportions of resilin in their thorax [15]. These pads are located at the hindlegs and associated with the trochanteral depressor muscles that actuate the jumps. Before each jump, the pads are compressed, and then their elastic recoils provide energy for the rapid trochanter movements powering the jump. While fleas perform their jumps just with the hindlegs, snow fleas (Mecoptera, Boreidae) use their hind and middle leg pairs for jumping. They feature four resilin-containing pads, one at each of the legs involved in jumping, and these pads are similar to those of fleas with respect to their locations at the legs and their function [19].

For the jumping mechanism of fleas it was proposed that the whole energy required is stored in the resilin-containing pads [15]. However, the results of recent estimations indicate that resilin alone often can provide only a rather small proportion of the energy that is necessary to fulfil the large power demands of fast leg movements involved in actions such as jumping [17]. Energy storage devices used for jumping in froghoppers (Hemiptera, Cercopidae) and planthoppers (Hemiptera, Issidae) and for jumping and kicking in locusts were shown to be composites of relatively hard and stiff chitinous structures and structures with large proportions of resilin [17–18,20]. It was suggested that a large proportion of the energy needed for jumping is stored within the hard and stiff chitinous structures, which (because of the stiffness of the material) likely requires only small amounts of bending and, therefore, only short muscle contractions [17–18,20]. The flexibility and elasticity of resilin are assumed to facilitate this mechanism by reducing the risk of fractures occurring within the stiffer material and by contributing to a rapid and complete return of the distorted energy storage devices to their original shapes [17–18,20]. It is conceivable that a large proportion of the energy storage devices existing in insects with fast leg movements, including those of fleas, feature composite architectures comparable to those described above.

The ability of resilin to store energy within jumping systems was shown to be also involved in an energy absorption function of a specific structure, called buckling region, that is present in each tibia of locust hind legs [76]. The buckling region is located in an area where the bending moment during jumping and kicking is high. When a hindleg slips during jumping or misses a target during kicking, this structure can buckle and thereby act as a shock absorber by dissipating energy that would otherwise have to be absorbed by other suctures such as the leg joints. The buckling region exhibits parts with large proportions of resilin that are assumed to contribute to the energy absorption and to the restoration of the original shape of the leg after buckling [76].

#### Flight systems: folds, tendons and microjoints

Resilin has already been found in various elements of insect flight apparatus, including tendons connecting muscles to pleural sclerites, wing hinge ligaments connecting the wings to the thoracic wall, prealar arms connecting pleural sclerites to the mesotergum, wing vein micro-joints connecting cross veins to longitudinal veins, regions of the wing membrane establishing a connection to the wing veins or defined patches within membrane cells, and cuticular layers within wing veins [2,5,21–22,77–79]. In these structures, resilin occurs either in the form of pure or nearly pure resilin (e.g., tendons) or mixed with varying amounts of chitin fibres (e.g., prealar arm), which tend to follow distinct directions or patterns and thereby influence the mechanical properties of the material [5,28]. All of these structures benefit to a more or less pronounced extent from the presence of resilin due to its low stiffness, high resilience, large and reversible extensibility, long fatigue time and ability of elastic energy storage and damping.

**Figure 5:**
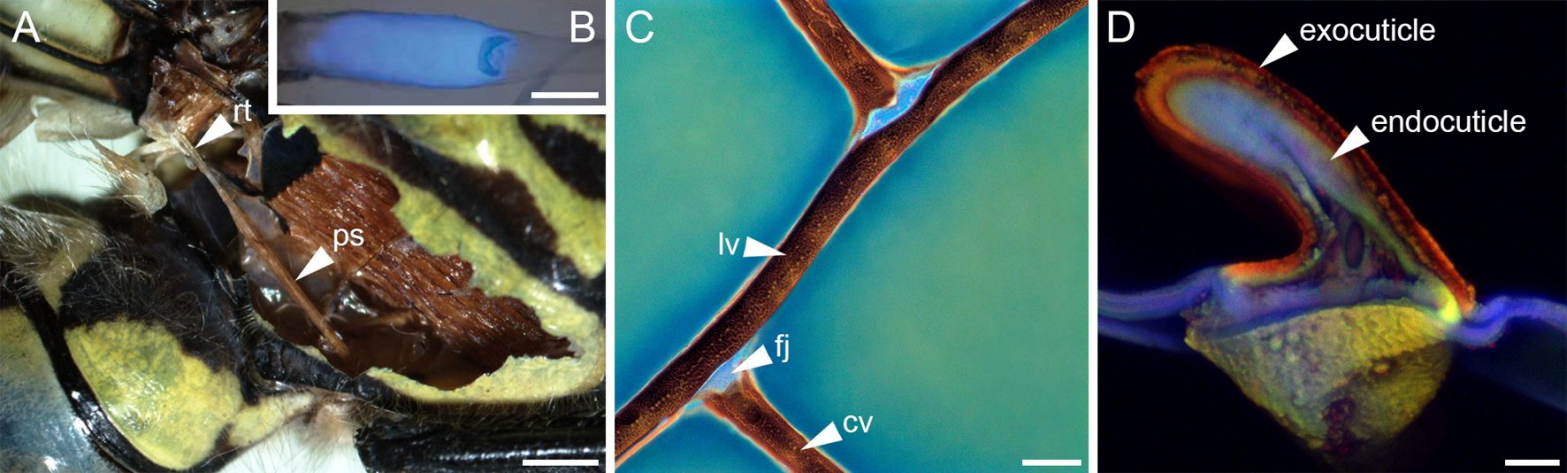
Resilin in flight systems of dragonflies. (A) Stereomicrograph depicting a resilin-bearing tendon (rt) of a pleuro-subalar muscle (ps) of the southern hawker (*Aeshna cyanea*). (B) Overlay of a bright-field micrograph and a wide-field fluorescence micrograph showing autofluorescence (blue) exhibited by resilin in a tendon of the genus *Zyxomma*. (C) Flexible wing vein joints (fj) joining cross veins (cv) to a longitudinal vein (lv) in a wing of the vagrant darter (*Sympetrum vulgatum*). (D) Cross section through a wing vein of *S. vulgatum*, revealing sclerotised exocuticle and resilin-bearing endocuticle. (C, D) Confocal laser scanning micrographs (maximum intensity projections) showing overlays of different autofluorescences exhibited by the exoskeletons. Structures with large proportions of resilin are shown in blue. Scale bars = 1 mm (A), 100 µm (B), 40 µm (C), 10 µm (D). (B) Adapted with permission from [13], copyright 2005 Nature Publishing Group. (D) Adapted with permission from [79], copyright 2015 John Wiley and Sons.

One of these flight system elements is a sausage-like swollen thoracic dragonfly tendon, which consists of virtually pure resilin and connects the pleuro-subalar muscle (which spans between the lower part of the pleuron and the subalar sclerite) to the subalar sclerite, which in turn connects to the axillary sclerites of the wing base [2] (Figure 5A,B). Together with the coxoalar muscle, it is assumed to control wing twisting (i.e., supination) during the upstroke by taking up wing movements and oscillating in length, while the attached pleuro-subalar muscle contracts slowly and tonically and keeps the tendon at a certain length and tension [5,80–81]. This is especially important for hovering and other refined flight manoeuvres [9]. It might also play a role in controlling excess wing motions during turbulent flows [81]. In the tendon, large reversible extensibility (e.g., over 250% in the forewing of the widow skimmer (*Libellula luctuosa*)) and a long fatigue time are of key importance for the functioning of this structure.

The wing hinge ligament of the forewing of locusts is located between the (meso-)pleural wing process and the second axillary wing sclerite [5]. In Odonata, Dictyoptera and Orthoptera, wing hinge ligaments exist in the form of thick, rubber-like pads (Figure 1A) and, as reported for locust structures, consist of a rather tough, mainly chitinous ventral part and a soft dorsal part, which can be divided in a region of pure resilin and a region containing both chitin lamellae and resilin [5]. The results of several studies suggest that wing hinge ligaments take up compressive as well as tensile forces and can contribute to (1) the storage of kinetic energy at maximum wing deflection, for example during the upstroke when the wing hinge ligament is stretched, and (2) wing acceleration during the downstroke by elastic recoil [5,28,82]. In other insects, such as Lepidoptera, some Coleoptera and some Hymenoptera, these ligaments are tough and inextensible, and elastic energy storage is likely provided by the rigid thoracic cuticle and the flight muscle itself [5,28]. Due to the fact that resilin has mainly been found in the flight apparatus of insects flying with synchronous flight muscles at low wing beat frequencies of less than 50 Hz and with inertial forces being larger than aerodynamic forces, it is assumed that its resilience might be too small at high frequencies [5,11,28,83]. However, there is still some controversy about the frequency-dependent behaviour of resilin and chitin–resilin composites and its function in the wing hinge ligaments of insects with high wing beat frequencies [5]. For example, some small wing hinge ligaments have been found between different sclerites in the genera *Calliphora*, *Bombus*, *Apis* and *Oryctes* [5,84]. So far, only a few studies have investigated the decrease in resilience with increasing frequencies in the dragonfly tendon, locust prealar arm and cockroach tibia-tarsal joint resilin [5,11,81,83,85]. Whether the partly pronounced differences in the decrease rate of resilience between different frequency ranges are due to different measurement techniques or are actually due to differences in the material composition, still needs to be elucidated.

The prealar arm is located at the front edge of the mesotergum and establishes a connection to the first basalar sclerite of the pleural thoracic wall via a tough, flexible ligament [5]. The basalar sclerite in turn is connected to the humeral angle of the anterior part of the wing base. The prealar arm consists of around 23% chitin and 77% resilin and is structured by alternating layers of resilin and chitin fibrils, with the fibrils continuing into the dark, sclerotised cuticle at its base [5] (Figure 1B). Due to the directional arrangement of chitin fibrils, the mechanical behaviour of the prealar arm is assumed to be dominated by the mechanical properties of the chitin fibrils during stretching and by the properties of resilin during bending and compression [5,11]. In contrast to the subalar muscle, which is involved in wing supination, the contraction of the basalar muscle causes wing pronation through the connection to the humeral angle via the basalar sclerite. During muscle contraction, the prealar arm is deformed and can be assumed to play a role in elastic energy storage.

Cross veins in wings of dragonflies and damselflies were shown to form either stiff, inflexibly fused joints or flexible, resilin-bearing joints to the adjacent longitudinal veins [24–26,78,86] (Figure 5C). The distribution pattern of different wing vein joint types on the dorsal and ventral wing sides in various species is quite diverse, but was found to follow phylogenetic trends probably related to wing morphology and flight behaviour [26,78]. In general, flexible wing vein joints, together with the overall corrugated design of odonate wings, are assumed to feature a larger angular displacement than fused vein joints and, as a result, to provide the wing with increased chord-wise flexibility, which promotes passive wing deformations such as camber-formation during the downstroke, and, thereby, to improve the aerodynamic and mechanical performance of the wing [24,26,78,86–87]. Moreover, resilin is important for reducing stress concentrations in vein joints [87]. Resilin is not only present in wing vein joints but also in the wing membrane directly abutting on wing veins and internal cuticle layers of wing veins (i.e., the endocuticle) [78–79] (Figure 5D). A flexible suspension of the wing membrane is suggested to allow larger strain and thereby to help preventing its tear-off from the wing veins [79]. Furthermore, the stiffness gradient in wing veins, generated by a stiff, sclerotised outer layer (exocuticle) and a soft, compliant, resilin-bearing inner layer (endocuticle) is assumed to reduce the overall vein stiffness and to improve the damping properties of the vein as well as to delay Brazier ovalisation and to enhance the load-bearing capacity under large deformations [79,88].

By artificially stiffening single flexible, resilin-bearing vein joints in bumblebee wings through the application of micro-splints (extra-fine polyester glitter glued with cyanoacrylate), it was experimentally shown that even a single resilin-bearing joint plays an important role in overall wing flexibility and vertical aerodynamic force production [89]. Ma et al. [90] found comparable resilin joints (e.g., the 1 m-cu joint) in wings of western honey bees (*Apis mellifera*) and assumed that they might play an analogous role in increasing the chordwise wing flexibility. Based on the distribution of resilin patches, wing veins, the occurrence of a flexible hook-mediated forewing–hindwing connection and observed wing deformations, they further suggested the existence of five flexion lines in one forewing–hindwing entity and assumed that these probably increase the cordwise flexibility and support camber formation. In addition, Mountcastle and Combes [91] demonstrated that a resilin-bearing joint at the leading edge (the costal break) in the wings of wasps plays a major role in mitigating wing wear by flexion along this joint when the wings hit an obstacle. This mechanism is especially important for wings with wing veins extending all the way to the tip because such a design endows a wing with more spanwise rigidity than, for example, bumblebee wings that lack veins at the wing tip [91].

The occurrence of resilin in several broadened vein patches as well as in membranous folding lines was described for fan-like dermapteran hind wings [22,92]. These structures help folding the wing into a wing package being ten times smaller than the unfolded wing. This package can then be hidden under the short sclerotised forewings. The four-fold wing folding can be achieved without musculature activity and is assumed to be driven by elastic recoil of the anisotropically distributed resilin on either the ventral or the dorsal sides of broadened vein patches in intercalary and radiating veins, supported by the resilin-bearing radiating folds that influence the folding direction [22]. Unfolding of the hind wings is achieved either by wiping movements of the cerci (e.g., in the European earwig (*Forficula auricularia*) and the lesser earwig (*Labia minor*)) or by wing flapping (e.g., in the earwigs *Timomenus lugens*, which has very long cerci, and *Auchenomus* sp.) [22,93]. Both unfolding mechanisms are supported by several wing stiffening mechanisms such as the mid-wing mechanism and the claval flexion line, which keep the wing unfolded in all species examined [22,93]. These mechanisms were found to play an important role both in the static unfolded state of the wing and during flapping flight, in which they help to inhibit an unfavorable folding of the wing [92]. Furthermore, the flexible resilin-bearing folding lines were found to not only serve wing folding but also act as flexion lines at which the wing flexes during flight, thereby supporting the generation of an aerodynamically favourable cambered wing profile [92,94].

In beetle wings, resilin was found to occur at the marginal joint, between veins that separate during folding, and along flexion lines in membranous areas, leading to the hypothesis that elastic energy storage by resilin can support wing unfolding also in beetle wings [21]. However, this can, if at all, only be a supportive role because wing unfolding in beetles was stated to be mainly achieved by scissor-like movements of the RA and MP1+2 veins via contraction of the Musculus pleura alaris and the basalar muscles, which is possibly supported by hydraulic hemolymph pressure [95–96]. Like in dermapteran wings, in beetle wings resilin most probably delays material fatigue in highly stressed wing regions and might further play a role in wing deformation during flight [22].

In wings of the urban bluebottle blowfly (*Calliphora vicina*), resilin is mainly present in the proximal part of the wing, predominantly in the form of resilin-bearing patches between veins [77]. The occurence of resilin coincides with the proximal distribution of the maximum spanwise bending stress at the beginning of each stroke cycle and suggests that the resilin patches reduce the risk of breaking near the wing hinge due to a decrease in peak stress in the rigid wing parts [77].

#### Attachment systems

The contact formation of insect adhesive pads on substrates depends on the ability of the pads to adapt to the surface topography. In this context, specific micro- and nanostructures can enhance the quality of the contact [97–101]. In the case of attachment on rough substrates, multiple contacts, being formed by some adhesive systems, provide great advantages [102]. The formation of multiple contacts, which contribute to an increase of the overall length of the total peeling line, is facilitated by a hierarchical organisation of the attachment structures [103]. It was shown that the combination of thin tape-like contact tips of hairs (setae) and applied shear force lead to the formation of a maximal real contact area without slippage within the contact [104]. This indicates that material flexibility is very important for the contact formation of adhesive pads. With a minimal normal load, flexible materials can create a large contact area between the attachment structures and the substrate. However, elongated structures that are too flexible have a low mechanical stability [105]. For example, if insect setae are too soft, they can buckle and collapse, and so-called clusterisation (or condensation) can take place [106–107]. As a result of this, the functional advantages achieved through multiple adhesive contacts can be strongly reduced [103]. Accordingly, the composition and the properties of the material of insect adhesive setae represent an optimisation problem. There is evidence that during the evolution gradients of the thickness and the mechanical properties of the setae have developed as a solution of this problem. The presence of thickness gradients, revealed by scanning electron microscopy, is well-known for various insect adhesive setae [97]. Recently, a gradient of the material composition, present on the level of each single adhesive tarsal seta, was shown to exist in the seven-spot ladybird (*Coccinella septempunctata*) [48] (Figure 2A–C). The material of the setal tip contains large proportions of resilin, while the base of the seta consists mainly of sclerotised chitinous material. Between the tip and the base, a pronounced material composition gradient was revealed by CLSM. This gradient is reflected by a pronounced gradient of the material properties: the setal tip is rather soft, whereas the setal base is relatively stiff. Both gradients were hypothesised to represent an evolutionary optimisation that increases the attachment performance of the adhesive pads when they attach to rough surfaces due to an efficient adaptation of the soft and flexible setal tips to the substrate and a simultaneous prevention of setal clusterisation by means of the stiffer setal bases [48]. Since this hypothesis is difficult to test experimentally using biological specimens, it was tested using numerical simulations [49] (Figure 2D–F). The results indicate that setae with long soft tips and rigid bases exhibit a strong adhesion but also a pronounced clusterisation (Figure 2E). Setae with rigid tips and soft bases have a low adhesion and a pronounced clusterisation (Figure 2F). Only setae with short soft tips and rigid bases feature optimal adhesion properties and simultaneously a minimum of clusterisation (Figure 2D), which confirms the hypothesis. Tarsal liquids produced by beetles are assumed to contribute to the adhesion efficiency of adhesive pads in the form of capillary interactions and cleaning effects. With regard to the resilin-dominated setal tips, an additional function is conceivable. As described above, resilin is only soft and flexible when it is hydrated. Accordingly, to keep the contribution of the large resilin proportions in the setal tips to the attachment performance of the adhesive pads on a high level, the hydration of the resilin must be maintained. It is imaginable that this is achieved by slowly evaporating tarsal liquids covering the setae and thereby keeping the resilin in the setal tips hydrated [48].

**Figure 6:**
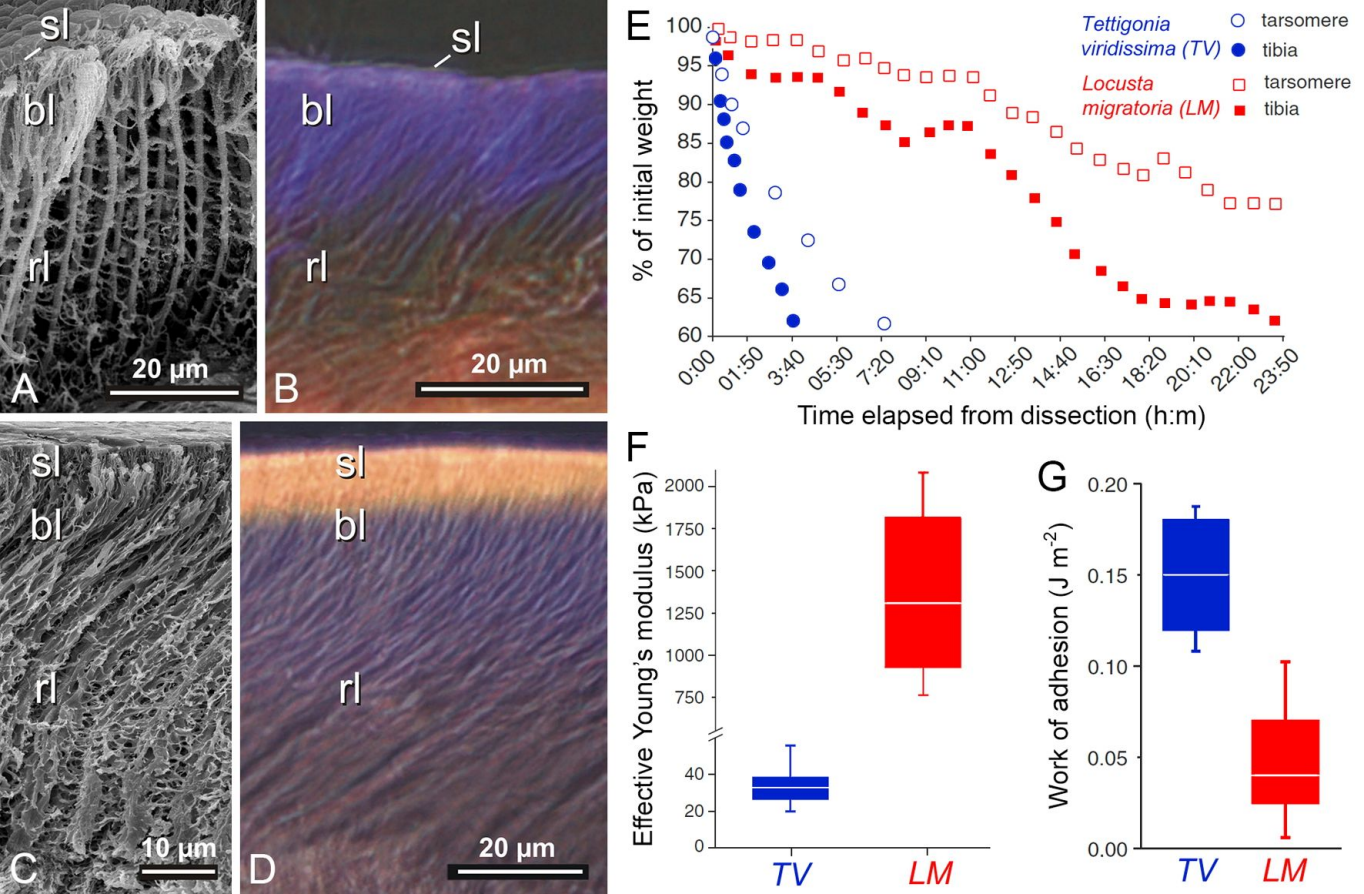
Material structure and properties of orthopteran adhesive pads (euplantulae). (A, B) Great green bush-cricket (*Tettigonia viridissima*). (C, D) Migratory locust (*Locusta migratoria*). (A, C) Scanning electron micrographs showing frozen, fractured, substituted, dehydrated and critical point dried pads. (B, D) Wide-field fluorescence micrographs showing frozen-cut pads. Structures with large proportions of resilin are violet/blue. sl, superficial layer; bl, layer of branching rods; rl, layer of primary rods. (E) Desiccation dynamics of tarsi and pieces of tibiae cut off the body in both species. (F) Effective Young's modulus of attachment pads of both species determined by means of indentation with a spherical tip radius of 250 µm. (G) Work of adhesion of attachment pads of both species measured by means of indentation with a sphere with a radius of 32 µm. In F and G, the ends of the boxes define the 25th and 75th percentiles, the lines indicate the medians, and the error bars define the 10th and 90th percentiles. (A–G) Adapted with permission from [23], copyright 2006 Springer.

The presence of material gradients has also been demonstrated for smooth attachment devices of insects [23]. Interestingly, the gradients revealed in smooth adhesive pads of locusts and bush crickets differ from those existing in the adhesive tarsal setae described above. The smooth pads contain a relatively soft core, which is covered by a stiffer layer. Accordingly, the direction of the material gradient is opposite to that in the adhesive tarsal setae, which can be well explained by the different pad architecture. Smooth pads feature branching fibres (rods) that form foam-like structures. The spaces between the solid structures are filled with fluid. Due to this construction principle, the pads are kept in shape. The fibres are terminated by a relatively stiff superficial layer that keeps the positions of the relatively long and thin fibres (and thereby the distance between the fibre tips) constant [23,108]. This pad architecture was studied in detail in two orthopteran species, the great green bush-cricket (*Tettigonia viridissima*) (Ensifera) and the migratory locust (*Locusta migratoria*) (Caelifera), whose adhesive pads generally have a similar structural organisation [23] (Figure 6A–D). Both pads possess a flexible resilin-containing exocuticle with fibrils that are fused into relatively large rods oriented in an angle to the surface. However, slight differences in the pad architecture exist. Adhesive pads of *L. migratoria* feature a clearly thicker superficial layer as well as a higher density of rods than those of *T. viridissima* (Figure 6A–D). In addition, indentation experiments revealed a higher effective Young’s modulus and a lower work of adhesion for *L. migratoria* pads (Figure 6F,G). The lower adhesive properties of *L. migratoria* pads can be explained by the larger thickness of the relatively stiff superficial layer, which likely reduces the adaptability of the pad to the substrate much more than the relatively thin superficial layer of the *T. viridissima* adhesive pad. The superficial layer is assumed to also protect the pad from desiccation as indicated by experiments showing that cut-off adhesive pads of *T. viridissima* (with the relatively thin superficial layer) lose water much faster than those of *L. migratoria* (Figure 6E). Consequently, the material gradient provides a combination of conformability to the surface roughness of the substrate (The compliant material of the pad contributes to the efficient contact formation with the substrate.) and resistance to the dry environment. Such pad architectures likely depend on the preferred environment of each species and are the result of trade-offs between different factors such as evaporation rate, stiffness, stability and adhesion.

#### Mouthparts

The first mouthpart-related structures containing resilin were already mentioned shortly after the description of resilin. In the respective studies, resilin was found in the salivary and feeding pumps of assassin bugs [109] (cited in [110]), [111]. Later, the findings were confirmed and complemented by additional information about the resilin distribution [51]. In these pumps, which enable the bugs to suck relatively large amounts of blood in a short time period and to inject proteolytic enzymes into prey or assaulters or to spit on the latter, the resilin-containing structures function as elastic spring antagonists to muscles. A similar function was described for resilin-containing structures present in the maxillipeds of decapod crustaceans [112]. The movements of the flagella of these mouthparts influence the water flow through the gills as well as over chemoreceptors located on the head, and thereby they importantly contribute to active chemoreception and to signalling by distributing urine odours. Each of the flagella is abducted by the contraction of a single muscle. Due to this abduction, a structure that contains relatively large resilin proportions and is located in the joint between the flagellum und the exopodite of the maxilliped is bent. After relaxation of the muscle, this elastic structure recovers its original shape and moves the flagellum back to its resting position.

In general, due to its very pronounced elasticity and fatigue resistance, resilin appears to be a very suitable material for exoskeleton structures that are typically intensively deformed for a rather large number of times during the lifetime of the organisms. A butterfly proboscis, for example, is tightly and spirally coiled when it is in its resting position [113]. For the uptake of food, hemolymph is pumped into the proboscis resulting in the generation of hydrostatic pressure that completely uncoils the proboscis [113–115] and strongly changes the shape of certain proboscis elements. During this process, dorsal parts of the proboscis are compressed. These parts contain relatively large proportions of resilin and act as springs that cause the recoiling of the proboscis when the hydrostatic pressure is removed [115].

A remarkable resilin-containing adhesive prey-capture device, which is formed by the elongated labium, exists in rove beetles of the genus *Stenus* (Staphylinidae). This prey-capture apparatus can be protruded towards a prey within a few milliseconds. When sticky pads (modified paraglossae), which are located at the distal end of the prementum, adhere to the prey, the labium is withdrawn immediately, and thereby the prey is transported to the mouth region of the beetle where it can be seized with the mandibles [116–118]. The sticky pads feature a surface that is subdivided into numerous terminally branched outgrowths. During the prey capture, these surface structures are completely covered by an adhesive secretion that is produced in special glands located in the head capsule and makes the sticky pads a hairy, hierarchically structured and wet adhesive system. Similar to the insect tarsal adhesive pads mentioned above, softness and compliance of the pad cuticle contribute to the generation of strong adhesive forces by the pads. The cuticle material of certain parts of the sticky pads contains large proportions of resilin providing flexibility and elasticity and enabling the pads to efficiently adapt to the surface of the prey items [118].

**Figure 7:**
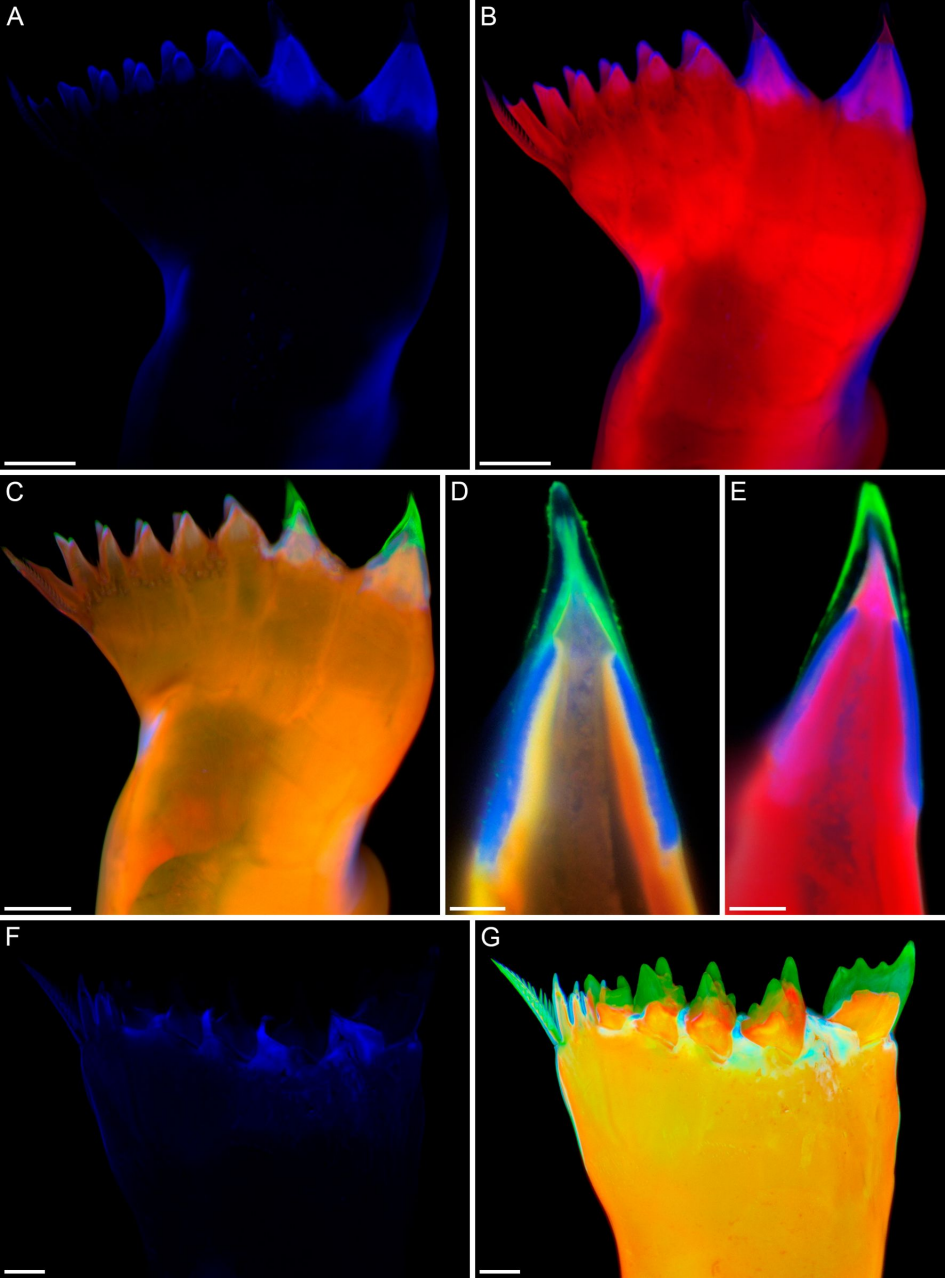
Morphology and material composition of mandibular gnathobases of copepods. Confocal laser scanning micrographs showing gnathobase structures of (A–E) a female of the copepod species *Centropages hamatus* and (F, G) a female of the copepod species *Rhincalanus gigas*. (A–C, F, G) Maximum intensity projections. (D, E) 1 µm thick optical sections through the largest tooth of the gnathobase. (A, F) Distribution of resilin. (B) Chitinous exoskeleton (red) and resilin-dominated structures (blue). (C–E, G) Chitinous exoskeleton (red, orange), resilin-dominated structures (blue, light blue, turquoise) and silica-containing structures (green). Scale bars = 20 µm (A, B, C), 5 µm (D, E), 25 µm (F, G). (A–E) Adapted with permission from [29]. (F, G) Adapted with permission from [30], copyright 2015 Elsevier.

Copepods are tiny crustaceans that inhabit nearly all aquatic habitats worldwide and are particularly abundant in the marine water column where they contribute large proportions of the zooplankton [119–120]. The diet of many of the marine planktonic species comprises relatively large fractions of diatoms (i.e., unicellular algae with silica-containing shells called frustules). Copepods use the gnathobases of their mandibles to grab and mince food particles. To be able to efficiently digest the diatom cells, the copepods must crack the frustules before the ingestion of the cells. The gnathobases possess tooth-like structures (called teeth in the following) at their distal ends [121]. In copepod species feeding on large amounts of diatoms, these teeth are rather compact and consist of complex composites that combine diverse structures and materials with a wide range of properties. Recently, the morphology and material composition of the gnathobases of two copepod species have been analysed and described in great detail [29–30]. The gnathobases of the calanoid copepod *Centropages hamatus* feature two larger and relatively compact teeth (Figure 7A–E). Each of these teeth possesses a chitinous socket, which is covered by a cap-like structure with a large resilin proportion. On top, another cap-like structure that is composed of silica is located. All other gnathobase teeth are smaller, contain no silica, are mainly chitinous and have tips with large resilin proportions. *C. hamatus* is omnivorous and feeds on, among other organisms, diatoms and protists. It is assumed that the large silica-containing teeth are used for feeding on diatoms. The silica makes these teeth stiffer and more mechanically stable and thereby more efficient in cracking the diatom frustules. In case the diatom frustules are too stable and the pressure acting on the tips of the siliceous teeth exceeds the breaking stress level causing an increased risk of crack formation in and breakage of the teeth, the soft and elastic resilin-containing structures are supposed to function as flexible bearings that can be compressed and thereby reduce stress concentrations in the tooth material and increase the resistance of the teeth to mechanical damages. Additional structures with large resilin proportions, located in the central and proximal parts of the gnathobases, are assumed to have a damping function that makes the whole gnathobases resilient and further reduces the risk of mechanical damage of the teeth. The smaller gnathobase teeth of *C. hamatus* are likely used to grab protists. In this context, the grip of the tooth tips is suggested to be increased by the soft and elastic resilin making the grabbing process more efficient.

The gnathobases of the calanoid copepod *Rhincalanus gigas*, a species whose diet mainly consists of diatoms, are characterised by five relatively large and compact teeth that possess a combination of different materials comparable to that of the silica-containing teeth of *C. hamatus* (Figure 7F,G). Each of these teeth has a silica-containing cap-like structure, which is located on a chitinous socket. At the base of the socket, the gnathobase exoskeleton features large proportions of resilin. Like in the silica-containing teeth of *C. hamatus*, these resilin-containing structures very likely function as compressible supports reducing the risk of mechanical damages of the teeth during feeding on diatoms with stable frustules. In general, the complex composite systems in the gnathobase teeth are assumed to have co-evolved within an evolutionary arms race together with the diatom frustules [122].

#### Reproductive organs, mechanoreceptors and compound eyes

**Figure 8:**
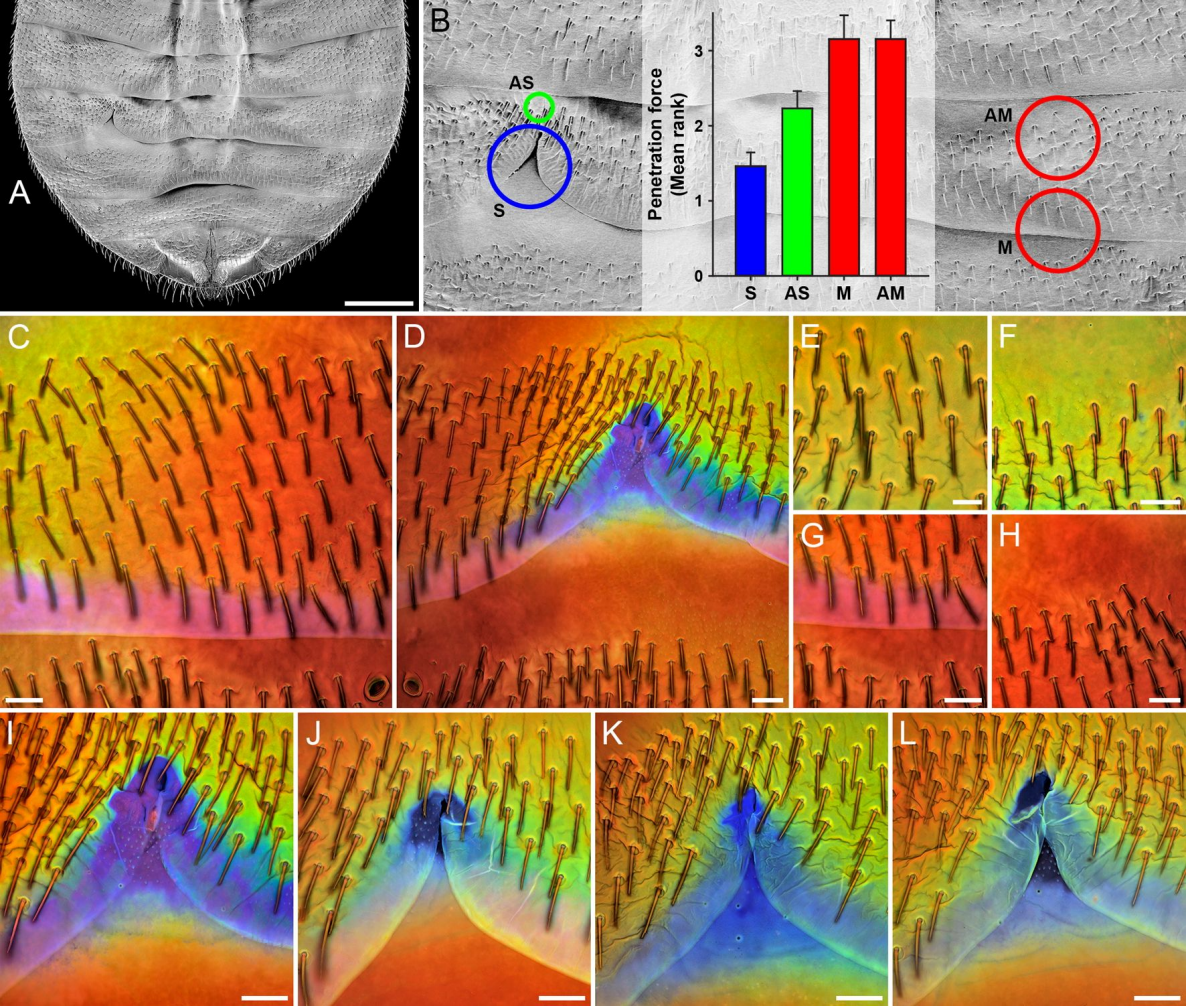
Material composition and properties of the ventral abdominal cuticle of females of the common bed bug (*Cimex lectularius*). (A) Abdomen overview (scanning electron micrograph). (B) Section of A indicating the locations of the spermalege (S) and three other cuticle areas called AS, M and AM, and penetration forces (mean ranks and standard errors) determined for these four cuticle sites. (C–L) Confocal laser scanning micrographs (maximum intensity projections) showing overlays of different autofluorescences exhibited by the exoskeletons. Blue colours indicate large proportions of resilin. (C, D) Autofluorescence composition of the cuticle in the left (C) and right (D) abdomen parts. The dominance of violet/blue autofluorescence (shown in blue) is restricted to the spermalege, clearly indicating that only at this site the cuticle contains large proportions of resilin. (E–H) Autofluorescence composition of the cuticle at the sites AS (E, F), M (G) and AM (H). The cuticle at M and AM consists mainly of sclerotised chitinous material, indicated by the dominance of autofluorescence shown in red, while the presence of large proportions of autofluorescence shown in green in the cuticle at AS indicates that the respective material consists mainly of weakly or non-sclerotised chitinous material. (I–L) Autofluorescence composition of the cuticle at the spermaleges of different one-week-old females, indicating variation of the extent of the resilin-dominated spermalege structures between females. Scale bars = 500 µm (A), 50 µm (C, D, F–L), 25 µm (E). Figure reproduced with permission from [126].

The mating of bed bugs represents a famous example of sexual conflict. During every successful mating event, the cuticle of the ventral side of the abdomen of the female is penetrated by the male with a cannula-like intromittent organ, and the male injects sperm and accessory gland fluids directly into the abdomen where the sperm migrate to the ovaries [123–124]. This traumatic insemination imposes survival costs on the females [124] but the females cannot avoid mating [125]. As a result of this sexual conflict, a female organ, the so-called spermalege, has evolved. In common bed bugs (*Cimex lectularius*), this organ is located on the right side of the ventral abdomen part where it is visible as a notch-like modification of the posterior edge of the fifth segment that exposes the subjacent intersegmental membrane and cuticle of the sixth segment (Figure 8A,B). A recent study revealed that the spermalege cuticle, by contrast to other cuticle sites analysed on the ventral side of the female abdomen, contains large proportions of resilin [126] (Figure 8C–L). In microindentation tests, the penetration force necessary to pierce the resilin-rich spermalege cuticle was significantly lower than that necessary to pierce the other cuticle sites [126] (Figure 8B). In addition, evidence for a significantly reduced tissue damage and hemolymph loss was obtained for piercings of the spermalege cuticle compared with piercings of the other cuticle sites [126]. The results suggest that the material composition of the spermalege cuticle has evolved as a tolerance trait that reduces the mating costs of both the female and the male: due to the softness of resilin the penetration is easier for the male and causes less wounding of the female, and after the withdrawal of the intromittent organ the elasticity of resilin causes a sealing of the puncture reducing the hemolymph loss and the risk of bacterial infection.

**Figure 9:**
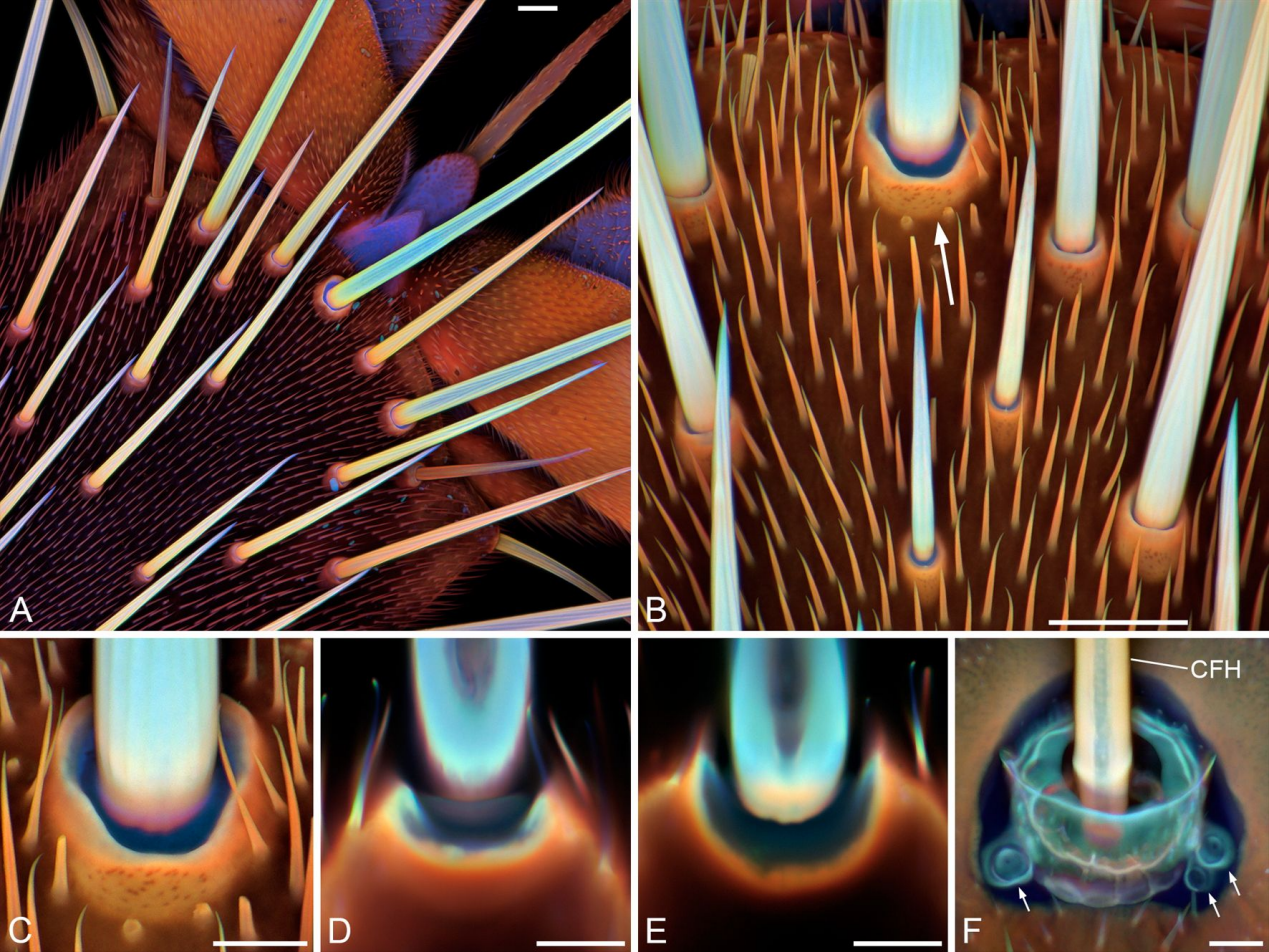
Resilin in mechanoreceptors. (A–F) Confocal laser scanning micrographs showing overlays of different autofluorescences exhibited by the exoskeletons. Blue colours indicate large proportions of resilin. (A) Dorsal view of the pretarsus of a third leg of a female drone fly (*Eristalis tenax*). (B) Dorsal view of a section of a second leg’s pretarsus of a male *E. tenax*. The arrow highlights a hair plate sensillum. (C) Larger view of the hair plate sensillum highlighted in B. (D, E) Confocal laser scanning micrographs showing 1 µm thick optical sections through the hair plate sensillum shown in C. (F) Cercal filiform hair (CFH) and associated campaniform sensilla (highlighted by small arrows) on a cercus of a female house cricket (*Acheta domesticus*). (A–C, F) Maximum intensity projections. Scale bars = 25 µm (A, B), 10 µm (C–F). Figure reproduced with permission from [10], copyright 2011 John Wiley and Sons.

Hair plate sensilla and campaniform sensilla are typical mechanoreceptors that are common in insect exoskeletons [127–129]. These receptors possess so-called joint membranes and cap membranes that are composed of large proportions of resilin [10,127,129]. On the dorsal side of the pretarsus of the drone fly (*Eristalis tenax*), for example, hair plate sensilla with rather long and relatively thick hairs are present (Figure 9A–E). The base of each hair is surrounded by a joint membrane that, due to its resilin-dominated material composition, is soft and flexible and allows movement and bending of the hair shaft resulting in a stimulation of the receptor. Because the long hairs project beyond and below the pulvilli of the pretarsus, they touch the substrate shortly before the pulvilli and likely have the function to indicate the upcoming contact between the pulvilli and the substrate. The cerci of crickets feature cercal filiform hairs associated with campaniform sensilla [128,130]. The latter and the bases and sockets of the filiform hairs are embedded in material that contains relatively large proportions of resilin [10] (Figure 9F) and, as mentioned above, very likely allow movement and bending of the shafts of the filiform hairs and the campaniform sensilla, which are very sensitive strain receptors (also called flex or displacement receptors), and thereby stimulate the respective dendrite of the sensory cell.

**Figure 10:**
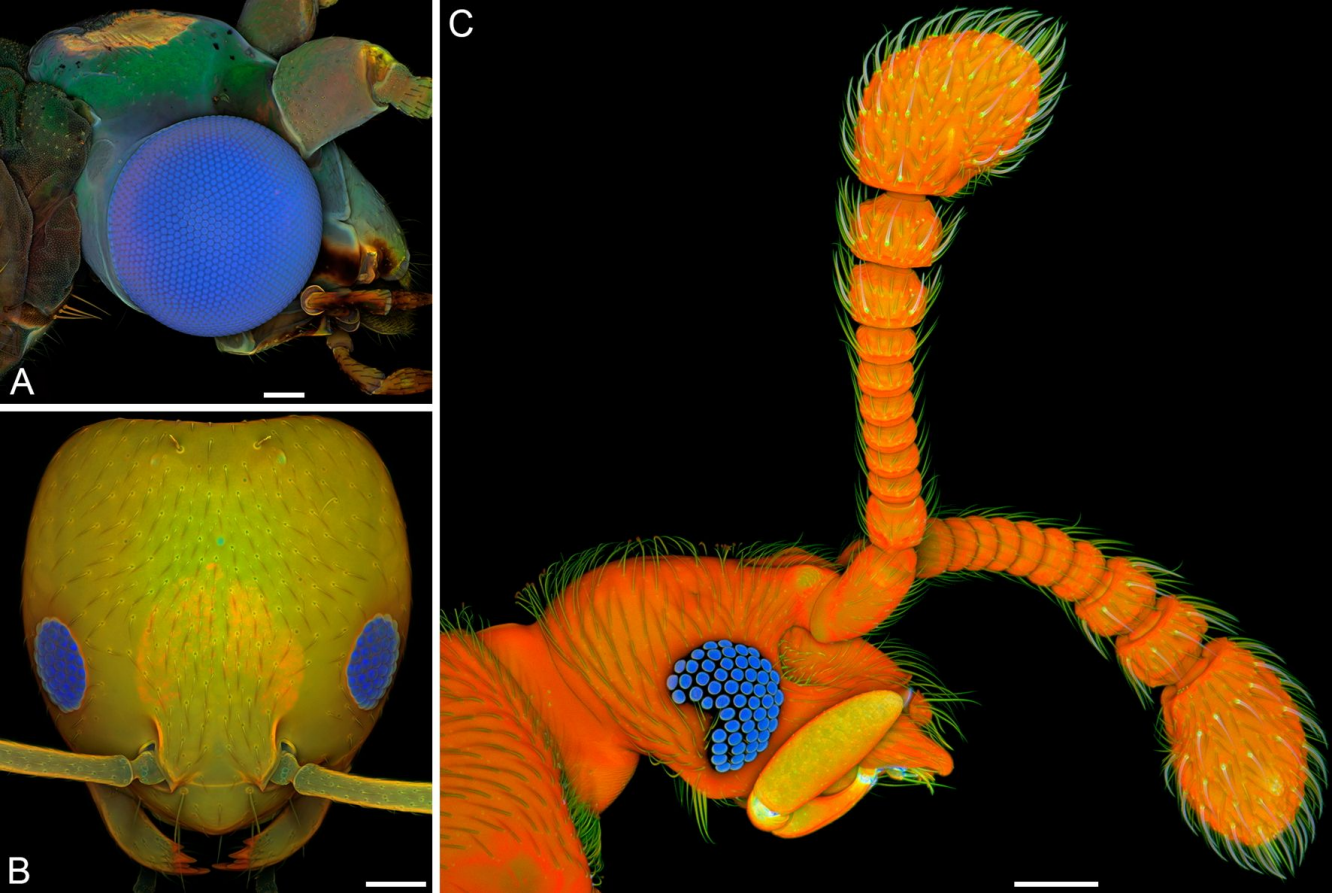
Resilin in compound eyes. Confocal laser scanning micrographs (maximum intensity projections) depicting overlays of different autofluorescences exhibited by the exoskeleton. Blue structures contain large proportions of resilin. (A) Lateral view of the head of a male green lacewing (*Chrysoperla carnea*). (B) Frontal view of the head of a pharaoh ant (*Monomorium pharaonis*) worker. (C) Lateral view of the head of a beetle of the genus *Circocerus*. Scale bars = 100 µm (A, C), 50 µm (B). (A, B) Adapted with permission from [10], copyright 2011 John Wiley and Sons.

The presence of large proportions of resilin (in part also described as ‘resilin-like protein’) in compound eye lenses has been described for different arthropods [10,131–134] (Figure 10). While other exoskeleton components are typically micro- and nano-structured, coloured and pigmented and, therefore, not suitable as material for optical elements, the pronounced transparency, the colourlessness and the amorphousness make resilin a perfect material for the construction of optical systems.

## Conclusion

Exoskeleton structures with large proportions of resilin are common among arthropods. This review demonstrates the broad range of resilin functions in various exoskeleton structures. Resilin facilitates flexibility and compliance, elastic energy storage, elastic recovery, fatigue and damage reduction, sealing and transparency and thereby makes the respective exoskeleton systems rather effective. Due to its remarkable combination of different properties, resilin is a highly efficient multi-functional protein. In addition, together with other compounds and materials, it often forms complex and powerful composites that combine the properties and benefits of the single components and are capable of performing rather specific and challenging functions. These characteristics have very likely been the reason for the evolution of the large functional diversity of resilin-containing exoskeleton structures in arthropods.
